# Taking care of myself in a different and broken world: self-care practices of adolescents on dialysis

**DOI:** 10.1080/17482631.2023.2171562

**Published:** 2023-02-01

**Authors:** Liliana Cristina Morales Viana, Edelmira Castillo-Espitia

**Affiliations:** a Department of Pediatric Nursing, Universidad del Valle, Cali, Colombia; b Department of Pediatric Nursing, Universidad del Valle Retired, Cali, Colombia

**Keywords:** Adolescents, dialysis, phenomenology, self-care, practices

## Abstract

**Purpose:**

Being on dialysis is a traumatic situation particularly during adolescence. Therefore, adolescents must have self-care support according to their special needs. The goal of this study was to describe the adolescents´ self-care practices when they are on dialysis. This paper reports only the adolescents´ selfcare practices based on the health care team guidelines but adjusted to their beliefs and habits.

**Methods:**

The methodology of the study was interpretive phenomenology. In-depth interviews were conducted with 15 adolescents; Smith´s IPA procedure was used for data analysis.

**Results:**

Being on dialysis meant to the adolescents living in a different and broken world. Self-care practices included: 1) coexisting with the dialysis slavery or being attached to a machine day or night, 2) struggling with the maddening thirst experience, 3) Deciding what, when and how much food to eat, 4) taking care of an alien that helps to survive, 5) taking the medicines when feeling in need of them.

**Conclusion:**

The adolescents tried to care for themselves adjusting their care activities to the health team´s recommendations. However, they also adjusted those practices to their beliefs and habits, which allows them to live with dialysis in a less traumatic way.

## Introduction

When chronic non-communicable diseases (NCDs) occur during adolescence, the impact is greater because they interfere with the growth and development of the adolescent (Bikbov et al., 2020; Slater et al., 2017). In recent decades there have been important changes in the epidemiology of NCDs in adolescence; the number of young people with this type of disease is increasing; Studies show that 13% to 27% of adolescents suffer from at least one NCD (Marani et al., 2020; Shorey & Ed, 2020). Kidney, cardiovascular and endocrinological diseases are among the main NCDs in adolescents (Krockow et al., 2019).

One of the most complex chronic diseases in adolescents is kidney failure, which has an increasing incidence and prevalence worldwide (Grewal et al., 2020). In Latin America, the incidence ranges between 2.8 and 15.8 new cases per year per million inhabitants under 15 years of age, with differences due to conditions such as the geographical distribution of the population and the socioeconomic situation of each country (Bikbov et al., 2020; Chávez et al., 2018). In children and adolescents with kidney failure, cardiovascular complications are the main causes of death (Modi et al., 2019). Treatment, particularly dialysis and kidney transplantation, contributes to high mortality; young people who receive these therapeutic actions have a mortality rate 55 to 150 higher than that of the general paediatric population (Chou et al., 2018).

Several models have been proposed and implemented for the health care of people with NCD. The most generic is the Model of the 5 A’s proposed by the WHO (World Health Organization, 2014); the most used is the Patient Centered Model; both models emphasize the promotion of self-care as one of the main axes (Fernandes et al., 2017; World Health Organization, 2014). Self-care has been extensively studied, but the omission of its psychosocial and sociocultural components is commonly noted in its approach since most studies have been carried out with a quantitative approach focused on adherence to treatment and how educational interventions influence and cognitive-behavioural in the clinical variables of the different NCDs (Adriaans et al., 2021; Bukhsh et al., 2018; Fleeman & Bradley, 2018; Gauci et al., 2021; Kim, 2018). From studies on self-care, it can be noted that in high-income countries the average adherence is below 50%, while in low-income countries the rates are even lower (Jose & Bond, 2021; Nsiah et al., 2021). Studies have been carried out mainly with adults, but it is adolescents who have the lowest adherence to treatments (Booster et al., 2019; Tan et al., 2021).

Given the complexity of both kidney failure treatment and self-care, it is valuable to listen to the experiences of adolescents concerning their self-care when in dialysis. The knowledge about how they live the self-care required can contribute to have an accompaniment of health personnel, particularly by the nursing professionals more focused on the adolescent´s reality, which would strengthen their self-care. For this purpose, an interpretive phenomenological study was carried out with adolescents living in several cities in Colombia. This manuscript only reports the findings on the self-care practices or activities the adolescents carried out, trying to follow the indications of the health team in charge of their care. The adolescents also had self-care practices that arose from their beliefs, customs, and initiative; these will be described in another paper.

## Materials and methods

### Design

The study methodology was based on some Heidegger phenomenology premises about the human being. People with chronic health events, such as adolescents suffering from kidney failure on dialysis, are constantly searching for the meaning of their everyday experiences to make sense of the world they live day by day. Interpretive phenomenology allows us to discover these meanings (Tuohy et al., 2013).

The premises about the person or human being (Leonard, 1994) help the researcher to perceive the persons participating in the study as self-interpreting beings, who have a world derived from their culture and history, for whom things are important, attractive, unpleasant, useful, and act according to this assessment in the present and the future; they have a body and bodily intelligence and they are in time, that is, they are constituted by their past, present and future.

The rigorous descriptive approach of phenomenology constitutes an adequate method to discover the experiences of self-care of adolescents on dialysis (Tuohy et al., 2013).

### Study Population

The sample were 15 youngsters, 9 men and 6 women, 15–19 years old (mean age 17.3 years). The selection criteria were: 1) age: 15–19 years; 2) to be or have been active in a dialysis program at least for six months at the moment of the first interview. In order to be active in the dialysis program, the adolescents should have manual or machine peritoneal dialysis each day at home or attend three times a week to the haemodialysis centre for a four hours section. The main disease that led to kidney failure were systemic lupus erythematosus and high blood pressure. At the time of the interviews, 12 of the youngsters were on haemodialysis in a dialysis centre and three on peritoneal dialysis at home (CAPD). Most of them started dialysis in their pre-adolescence and adolescence and had been on dialysis for 1 to 5 years; two adolescents received kidney transplant, 5 and 2 years before the interviews. Only two girls had experiences with haemodialysis all the time, one of them because of fear having an anormal abdomen and the other because haemodialysis would give her more time for other daily activities.

In qualitative research, data should be collected to get saturation according to different authors (Guest et al., 2020; Vasileiou et al. 2018) . However, this criterion is difficult to accomplish due to the complexity of the human phenomena. Mayan (Mayan & Introductoria, 2001) suggests the researcher should collect data up to the moment something important and new could be said about the study phenomenon. The number of adolescents was determined by the following reasons: 1) the data provided by the 15 adolescents, who accepted to participate in the study and met the selection criteria, was considered by the researchers as appropriate to describe the adolescents’ selfcare when in dialysis, especially their daily practices at home. 2) the data collection process was very time consuming and lengthy due the adolescents prolonged hospitalizations that interfere with the interviews; 3) the researchers had to finish the study according to specific date due to academic and funding reasons. The convenience sampling technique was used to choose the adolescents, according to the established selection criteria. The adolescent names used in this paper are fictitious. How the adolescents were contacted is described in the following section.

### Data Collection

The main researcher visited the waiting rooms of the Renal Units to contact adolescents in dialysis; through the “snowball” strategy she obtained contact information about other adolescents. Potential participants were also sought through service colleagues who cared for adolescents with kidney failure on dialysis in ten cities in Colombia. Finally, 25 adolescents and/or family caregivers were contacted, of which 4 did not agree to participate and 2 did not meet the selection criteria. The first contact with the adolescents was made in the waiting rooms of the renal units before or after a dialysis session. In agreement with the adolescent and her/his legal representative, a meeting was scheduled to provide them with information about the study, request her/his participation and obtain informed consent/assent.

The data was collected mainly through in-depth interviews lasting 45 minutes to 1½ hours, conducted by the main researcher, and analysed with the other researcher. Additionally, after the first interview, nine adolescents were given a USB memory device so that they could record aspects of self-care that they wanted to share with the researcher. The device was only given to adolescents who had access to a computer. In the USB device, they could deposit photos, videos, audios, or writings related to self-care. Only two adolescents provided information on the USB device.

Most of the adolescents were interviewed twice at their homes and a third by telephone; Only with one adolescent the three interviews were by telephone since she was in isolation for an infectious-contagious disease. Data collection lasted approximately 1½ years due to the multiple and prolonged adolescents´ hospitalizations, which continually forced to postpone the interviews appointments. The interviews were recorded and transcribed verbatim by the main researcher and by a person trained for this purpose; in this case, the main researcher reviewed the transcript to ensure that it corresponded to the recording.

### Data Analysis

The Interpretive Phenomenological Analysis (IPA) proposed by Smith (Smith & Shinebourne, 0000) was used for the interpretation of the data. According to Smith, the first step of the analysis is data immersion. To do this, the main researcher read iteratively the interviews transcriptions to get familiarized with each participant´s data. At the same time, the main researcher wrote comments on the transcriptions regarding aspects that seems interesting and significant in each data fragment. Then, she identified concepts in the data that were discussed with the co-researcher to identify the themes. The themes were listed on a paper sheet and both researchers look for theoretical connections between them and identify subthemes. Adolescents ´self-care practices were identified as a theme that included the following subthemes: Living Attached to a Machine during the day, Living an Overnight Slavery, Struggling with the Maddening Thirst Experience, Deciding What, When and How Much Food to Eat, Taking Care of an Alien that Helps to Survive, Taking the Medicines When Feeling in Need of Them. Then, both researchers did the subthemes description; during this stage, continue the data analysis process which becomes more expansive as the subthemes were explained, illustrated, and nuanced.

### Ethical Aspects

The study was approved by the Institutional Human Ethics Review Committee (CIREH, its Spanish acronym) of the Health Sciences Division at Universidad del Valle, Cali, Colombia according to the Certificate No. 020–017. The adolescents and their legal representatives were informed of the objectives of the study, the activities they would carry out as participants, the study would not cause them expenses, nor would they obtain financial benefits from it. They also were guaranteed the confidentiality of the data, informed of their rights, and signed the informed consent/assent form.

## Results

The adolescents experienced self-care in various ways, performing self-care practices according to two patterns. The first pattern included activities carried out following the guidelines of the health care team in charge of their care such as nephrologist, nurse, nutritionist, psychologist, and social worker but adjusted to the beliefs and customs of the youngster. The second pattern comprised activities according to the adolescent´ initiative, history, habits and beliefs.

The adolescents perceived entering the dialysis program as living a different life, with new care and restrictions, very different from those they were used to. They felt their world had been broken, by having dialysis as the option to be alive. They perceived ruptures in their daily life at home and as a student, in autonomy, in controlling time, and in eating habits and choices. The youngsters felt from the moment they entered dialysis their life should be planned solely in light of the disease and dialysis. The daily self-care practices performed by the adolescents in their transformed world are described below.

### Living attached to a machine during the day

The youngsters were quite aware dialysis is paramount in the treatment of renal failure; they had learned from their own experiences the serious health complications they faced when they did not undergo dialysis. This dependence on dialysis made them feel they should “live attached” to the dialysis machine or “be slaves” of it. This slavery feeling was independent of the dialysis modality. Teenagers felt they were attached to the machine too much time, which prevent them for going to school, being with friends and peers, having recreational activities. However, for some of them, undergoing dialysis was the most important self-care practice, because despite the difficulties and sadness it caused them, the dialysis kept them alive and helped them to prepare for kidney transplantation. Only one young man, Daniel, diagnosed with a hypoplastic kidney, decided to free himself from the dialysis and did not follow its protocol. Daniel gave priority to his job since he worked occasionally so he did not attend dialysis if the day they requested his services coincided with it.

According to the protocols of health institutions, haemodialysis is performed three or four times a week, for 4 hours, in the morning, afternoon, or evening. Youngsters who were in the morning schedule began their daily routines quite early; some got up at 4 in the morning, others at 5, depending on the distance from their homes to the dialysis centre. This schedule represented a great effort, as can be seen from the statement of one of them: “ … *and thinking about the difficult things again … from the early morning, it is difficult to go to dialysis to get up at five in the morning, one there warm and having to get up and it is cold, that is **very** difficult”*.

Some adolescents lived two or three hours by car from the haemodialysis centre (in cities other than the dialysis centre), which increased the feeling of slavery, boredom, sadness and anguish. They also referred the discomfort produced by dialysis, including cramps, headache, lack of energy, chills, nausea and sometimes vomiting. In addition to the great physical discomfort, they felt anguish and fear. They used various strategies to deal with these feelings, such as taking antihypertensive medications which made them drowsy at the beginning of the session, so they slept at least three of the four hours of the session. Camila did homework or read books, and several young people listened to music.

Another self-care practice was knowing their *dry weight*, a measure of body weight that serves as a reference to calculate the fluid amounts that are extracted during the dialysis session. Bearing in mind the dry weight figure was very important for the adolescents because if a fluid amount was extracted below it, they suffered muscle cramps, headache, nausea, or vomiting, and post-session fatigue. They had learned to be aware of their blood pressure figures because of the unpleasant symptoms that appeared when it began to drop. In this regard, a young man declared: *“The nurse and I agree on the fluid amount that she is going to get out of me because I know the amount, I don’t get those cramps that are so hard. I tell you one thing, between the nurse and the patient there must be a connection”*.

Only three adolescents were in school at the time of the interviews, they reported the dialysis time prevented them from attending school, and they prioritized their health over school. Having to prefer dialysis over school caused them sadness, anger, uncertainty, and increased the feeling of slavery from the dialysis machine, the health institution, and their parents or family caregivers. They considered dialysis affected the quality of their life and aspirations because their plans to study, to get a job and have a family of their own were postponed.

### Living an overnight slavery

Peritoneal dialysis should be done every day, during the day or at night; it is usually done at night. The duration of nocturnal dialysis is on average 10 hours, with an additional hour for connection and disconnection to the machine. This modality requires more time than haemodialysis (an average per week of 12 hours for haemodialysis vs 77 hours for peritoneal dialysis).

Of the 15 adolescents three were on peritoneal dialysis at the time of the interviews. They described in detail the procedure from connection to disconnection of the machine. They were emphatic in stating the main care with this modality is good hygiene at home and in the room where they sleep, with the dialysis machine, personal hygiene, and especially hand washing when performing peritoneal dialysis. They had suffered multiple episodes of peritonitis, which made them meticulous about hygiene; they knew any mistake in cleaning would yield a new episode of peritonitis.

Only Paola was on dialysis during the day. For her, the ideas of slavery and malaise were similar to other youngsters, but her dialysis was a great obstacle to work. Her family of indigenous peasants everyday work the land for their sustenance while she was on dialysis so she could not work with them, which caused her sadness, anger, anguish, and impotence. In an interview, she expressed a wish to die because the disease and dialysis did not allow her to work, and this made her a heavy burden for her and her family.: “ … *because a sick person cannot, cannot work the land, I would rather … . I think that it would be better to die!”*.

Samuel felt the slavery of dialysis had significantly altered his relationships with peers, especially with his girlfriend. He had not been able to be with her at night so he had to change his visiting routine for the daylight hours. The perception of slavery was more accentuated in this group of youngsters because *“they lived counting the hours to be connected”*, and they perceived the morning and afternoon hours much shorter than those at night when they were in dialysis.

### Struggling with the maddening thirst experience

All youngsters in the study pointed out the importance of controlling the fluids amount they had to drink each day, but for most, it was something impossible to do. They recognized this practice as important because they generally felt very ill when they had fluid overload: they had felt tachycardia, dyspnoea from small and large efforts, fatigue, and many times they were hospitalized due to pulmonary oedema.

The reasons to meet this difficult recommendation, which they perceived as especially overwhelming and impossible to achieve, were: a) Difficulty in measuring the fluids they consumed daily, particularly when they left their homes, even though they intended to do so because they did not have the elements or the way to measure them, and also they were embarrassed with their peers; b) Thirst exceeded their capacity for self-control due to the anguish it caused them. Most of the youngsters could not live with 800 cc per day, therefore they did not comply with this recommendation: “*Thirst is a weakness in the life of people who have kidney failure and are on dialysis, it cannot be controlled; the experience of thirst is very difficult and distressing, often the willpower to control thirst is depleted”*.

Despite that thirst was difficult to control, most of the youngsters tried not to exceed the recommended volume and used strategies such as sucking on ice at times of maximum thirst, eating acidic fruits, and sucking on ice cream made from acidic fruit juices. However, all agreed that these recommendations did not serve to reduce thirst because sometimes they sucked so much ice that it was better to have a glass of water. Three self-care patterns were distinguished regarding fluids restriction: 1) strict adherence to the recommendations of health personnel despite the discomfort caused by thirst; 2) adjustments and modifications to the recommendations of the health team; and 3) non-adherence to the recommendations and drinking fluids until thirst is quenched.

Those who followed the recommendations of the health team, at the beginning of the day, put 800 cc of water in a bottle, and each time they consumed some liquid they took the same amount out of the bottle. This strategy allowed them to know the amount of liquid they were consuming because they could visualize what they had drunk or what they could still drink. Another strategy, suggested by health personnel, consisted of drinking liquids using small glasses, using a small personal cup. Luisa weighed herself several times during the day to confirm that she had not exceeded her fluid intake.

The main strategy used by the adolescents to control the fluids amount was calculating the amount of fluids to drink by trial and error: they drank small additional amounts in hours of maximum heat and more fluids during the days they attended dialysis. Because they felt very thirsty after dialysis, some drank up to two glasses of fluids after therapy, even though they knew the possible consequences of doing so: *“You can’t control yourself when you’re on dialysis; you want to drink, drink and drink liquids. The feeling is as if you were being kidnapped and you haven’t been drinking liquids for a long time. There are times when I have been so anxious about thirst that I think ‘I’m going to drink water until my thirst is gone.’ Ah, one is going to die of something!”*.

Other adolescents drank liquids according to their thirst; that is, they did not have any type of restriction with the fluid intake. Some never had complications from ignoring this recommendation, while others had multiple hospitalizations. The nephrologist prescribed an additional session of dialysis to the adolescents who had complications from having fluid excess. This and repeated hospitalizations forced them to decide to significantly decrease their fluid intake.

### Deciding what, when and how much food to eat

All the adolescents acknowledged the difficulties in living with dietary restrictions; however, they recognized the modifications in the diet as a daily care practice that represented a great challenge. As they did with the restriction in the fluid consumption, they made adjustments in the diet according to their criteria, such as eating “*occasionally*”, or eating “*few quantities*” of forbidden or not recommended foods. The “*occasionally*” strategy consisted of eating prohibited foods from time to time: three times a week, four times a month, three times a month, each month, and for one of the participants once a year. For obvious reasons, some had more complications from doing this. However, this strategy helped them to live better with the diet restrictions.

For occasionally eating prohibited or non-recommended foods, everyone had the day of dialysis as the reference. Some ate them one day before dialysis, thinking that the next day the dialysis would “sweep” toxins and excesses of potassium, sodium and phosphorus from these foods; others ate them on the day of dialysis considering that that day the body tolerated excesses better because it was “*toxin free*”. Others ate the forbidden foods immediately after a dialysis session for the same consideration; that is, believing that it would be less harmful because after dialysis the body was similar to a healthy body: “*I am judicious, I take care of myself, but I do eat … for example, I eat seafood three times a month, but one day before dialysis; Thus, when they take my tests, neither phosphorus nor potassium is high. Same as pizza and dairy, but those are out there … ¡Very little!”*.

Some adolescents decided to eat “*few quantity*” of all kind of food. Those who used this strategy believed that food by itself was not harmful because those were sometimes forbidden and sometimes recommended to consume during medical appointments. Thus, eating a small amount of food was adequate so as not to suffer so much and to better support the diet they should have. The results of the laboratory tests confirmed the perception that the food was not harmful. In addition, adolescents considered the health team recommendations were exaggerated and if they accepted them, the offer of food for their nutrition would be reduced to two or three items: *“it makes me angry with the food, almost everything is forbidden there in the renal unit; so, what is one going to eat? I say that you can eat everything, but in small quantities, even if the nutritionist says no, I say that you can, but in small quantities”*.

Adolescents perceived their family as a great facilitator in complying with food restrictions, mainly because mothers, grandmothers, or aunts prepared the food according to the recommendations. In addition, in several families, all the members ate the same foods as the adolescent, which was especially motivating for the youngsters. On the other hand, those who used trial and error to live with the situation, when they noticed a specific food did not affect their health, they felt tranquillity, hope, and some normality in their lives. However, they thought that this was temporary because, even when they were transplanted, dietary limitations would be part of their treatment and, therefore, of their lives.

### Taking care of an alien that helps to survive

For the study adolescents, the catheter and arteriovenous fistula (AVF) were something grotesque that deformed their bodies, they were ashamed of so they tried to hide it under their clothes. Yet, they knew it was something necessary to live until they were transplanted. Given the indispensable nature of these devices, most youngsters referred to their care as essential. The practices they performed with the haemodialysis accesses are described below in the following order: haemodialysis catheter, peritoneal dialysis catheter, and AVF.

Self-care practices with the haemodialysis catheter were aimed at avoiding catheter infections and displacement. Catheter infections had been the cause of multiple and prolonged hospitalizations for the study adolescents so they were very afraid of catheter infections because those had seriously affected their health, and some had been several times in a coma, hospitalized up to three months in ICU. The fear of catheter infections was related to pain, boredom, loneliness, sadness, and anguish over hospitalizations. Some had had up to 15 catheter changes and, therefore, had multiple scars especially on the neck, chest, abdomen, and back. These scars reminded them of the disease they lived with; they felt ashamed and angry because the scars were visible and permanent reminders of the disease that would never fade, on the contrary, it might increase over time.

The main self-care practice to prevent or lessen infections related to the catheter was to avoid getting it wet. Before the daily body bath, some adolescents placed a small towel on top of the catheter and then taped a plastic bag over it. Hellen used a different approach: she placed a piece of self-adhesive plastic paper over the dressing covering the catheter. She also used the towel and plastic technique, but it did not work well for her. With the self-adhesive plastic paper, she managed to better protect the catheter and avoid hospitalizations and surgeries for infections.

Taking the daily bath was modified to prevent the catheter from getting wet. Most youngsters exposed the lateral half of the body to water (the side opposite to where the catheter was housed), soaped and rinsed it, and the side of the catheter was cleaned with wet cloths. Women had to wash their hair with another person´s help or they had to do it in a place other than the shower. Of course, this produced discomfort and sadness to the girls. Only Laura covered the catheter before entering the shower, as she had been instructed in the renal unit, and she washed her entire body in the water, otherwise she felt she had not taken a bath. She reported taking her bath this way, caused her several hospitalizations for catheter infections, ranging from local infections to sepsis that led to coma and prolonged hospitalizations. Another strategy to avoid wetting the catheter was to avoid bathing in pools and rivers, something particularly difficult for those who lived in hot climates.

Not doing physical exercise was another way to avoid getting the catheter wet, since the sweat moistened the dressing that covered the catheter and it came off. Due to pressure from their peers, some youngsters carried out physical activity at school, being aware that after finish in it they had to change the dressing, with the risk of displacing or contaminating the catheter; sometimes they attended the health institution to do the catheter dressing change. Covering the catheter with clothing was another self-care practice to avoid infection. Clothing should be clean, loose-fitting and made of cotton to reduce sweating.

As a self-care practice to avoid the catheter coming out, the adolescents tried not to fall or make physical efforts such as lifting heavy objects with the arm on the side where the catheter was located. When coming into physical contact with other persons, the teenagers tried to maintain a safe distance to avoid direct contact with the catheter; they also avoided sleeping on the side of the catheter or making sudden movements with the limb on the side they had it on. Youngsters with a hepatic-catheter as dialysis access were especially careful about the practices to avoid accidental displacement.

Self-care practices with the peritoneal catheter were aimed at avoiding peritonitis and its displacement. The adolescents avoided peritoneal infections by being meticulous about hygiene when connecting and disconnecting to the dialysis machine; they were certain any mistake could represent a new episode of peritonitis. Before the connection they did handwashing, tied their hair or used a hat to avoid contact with the catheter, wore a mask and washed their hands again.

Regarding the connection and disconnection of the machine, they pointed out the aseptic technique was central. Cleaning of the catheter hole was also mentioned among their self-care practices. This was done in two moments: a) immediately after disconnection from the dialysis machine, with a soap solution provided by the renal unit and with clean water, and b) during the daily body bath using the same soap and water they used for the body.

Adolescents on peritoneal dialysis permanently wore a bellyband, a cloth piece that is placed around the waist to store the catheter to protect it from infection or accidental displacement. The bellyband had to be washed every three days with mild soap, ironed, and changed it every day; some adolescents changed it every two days. Wearing the bellyband affected only the girls’ usual dressing. For one boy, the bellyband was “a good buddy”.

The youngsters considered hygiene as the main self-care practice when they are on peritoneal dialysis. Proper hygiene should be done during dialysis with the catheter hole, the dialysis machine, and the place where it is performed, as well as the whole body and clothing. They perceived more difficult to live with peritoneal dialysis than haemodialysis because there is a greater susceptibility to infections and long hospitalizations for peritonitis.

Avoiding displacement and catheter detachment was equally important; even though they knew from their experiences that the accidental detachment is infrequent, they carried out activities to prevent it such as avoiding sleeping on the side of the catheter, pulling it, lifting heavy objects and permanently wearing the bellyband.

Self-care practices with the AVF sought to avoid the physical deterioration of the fistula, especially the occurrence of an aneurysm. Therefore, the adolescents did activities such as caring for the skin that covered the AVF by washing it during the daily bath with mild soap and applying moisturizing cream, avoiding having blood drawn from the AVF or taking the blood pressure in the AVF arm, and avoid exerting the arm of the AVF.

### Taking the medicines when feeling in need of them

The adolescents had to start taking medication from six in the morning which compromised their sleep hours; in many cases taking the medicines coincided with the time to get up. Taking medications ended between midnight and one in the morning, and for some the last dose coincided with bedtime. Taking into account the number of pills the adolescents took per day (13–21 tablets daily), they had to take pills every two, three, or four hours. The times and intervals to take the pills made the youngsters very sad and reaffirmed the idea of slavery due to illness and dialysis.

Due to the great dislike the youngsters experienced with the medication schedules and the great number of pills, some adolescents adjusted the therapy according to their criteria, taking only the medicines they believed were necessary. In this sense, they felt autonomous since each youngster knew him/her very well and could decide which medicines and when to take them. For example, if they had symptoms of increased blood pressure such as headache, increased heart beats or heat, they took the antihypertensive pills. Some youngsters learned to take their blood pressure, and when the figures were normal, they did not take the antihypertensive pills. Adolescents also did not take those medicines they considered less important, such as vitamin D_3_ and calcium. However, they never stopped taking the medicines they considered most important, such as anticonvulsants. Several young men had seizures due to high blood pressure; this fact motivated them to comply with the treatment.

Despite dissatisfaction with drug therapy, adolescents used different strategies to comply with this self-care practice. The most used strategies were: to set alarms on the cell phone with the time and name of the medicine they had to take, to use pillboxes to avoid forgetting, especially when they had to travel to another city. Only one girl used the pillbox daily at home, placing the pills at the times and on the corresponding days for a week; when she had to leave home for a short time she used *“a little pillbox*” with the quantity she had to take: *“There are too many, but so as not to forget on my cell phone I have the alarm. Also, in the alarm, I put what tablet I have to take. If, for example, I have to go out, I take my tablet in a container and when I go out, I take it, or when … or sometimes to not carry that bottle I put the tablet in a pillbox, and since it is small, I put it in my pocket, and I walk with that”*. For this girl was important to hide the pills when going out.

Only Valentina used a poster she had at home with the pills names and hours. Checking the physician´s prescription was another strategy to remember to take medications, particularly for men. Tomás made a special “care guide” with essential information about his medicines.

Some youngsters did not take the medications because they thought it was a waste of time since the disease would not go away regardless they would do take the medicines properly. Samuel “*pretended to be crazy*” (meaning not to pay attention to) about the medicines, even though his mother was watching him to do so; when she asked him, he told her he had already taken them, although he had not. He suffered from SLE and knew this disease is incurable so he did not see the purpose of taking medications even after having the transplant, the lupus would possibly deteriorate the new kidney.

The shame that adolescents felt for taking medicine in the presence of others outside home, especially their peers, led them to fail to comply with drug therapy since taking medicines was a confirmation they were sick.

A young man had a particular practice: he replaced antihypertensive medication by immersing his feet in ice water (a strategy recommended by his grandmother). He took the antihypertensive medication only when this practice did not lower his blood pressure.

## Discussion

Self-care has been fundamentally approached as adherence to treatment (Adriaans et al., 2021; Bukhsh et al., 2018; Fleeman & Bradley, 2018; Gauci et al., 2021; Kim, 2018); adherence for adolescents with NCD is below 50% (Booster et al., 2019). The findings of the study are consistent with this report since the youngsters partially followed the recommendations of the health team, given that they carried out actions based on those recommendations, but modified them according to their beliefs and habits and by trial and error, which undoubtedly affected the adherence to the prescribed treatment. Each self-care practice is discussed below.

### Living attached to a machine during the day or at night

The meaning of the dialysis for the adolescents regardless its modality as a slavery from a machine is a relevant finding in this study. Despite this sense of slavery, most of the adolescents attended haemodialysis sessions or underwent peritoneal dialysis as prescribed; only one teenager prioritizes his job over haemodialysis. The adolescents accepted this slavery because dialysis was their only way to be alive. Despite, the study sample is not statistically representative, since it is not a quantitative study, the main researcher’s contact with these adolescents for more than a year allows us to affirm dialysis adherence was very high among them. This finding contrasts with studies such as those by Ozen, Cinar, Aski, Mut, Turker (Ozen et al., 2019), Chua y Warady (Chua & Warady, 2011) and Miyata et al. (Miyata et al., 2018) that reported adhesions below the 50%.

The literature reports most adolescents with kidney failure on dialysis suffer from depression due to the disease and its treatment (Kogon et al., 2019). In the present study, none of the youngsters reported to have a depression diagnosis by a psychiatrist. However, all experienced negative feelings regarding dialysis such as boredom, sadness, anger, anguish, permanent fear of death. This study did not delve into these emotional aspects, which could suggest a sub-diagnosis of depression in these adolescents. In addition, the reported loss of appetite, drowsiness, or insomnia associated with dialysis deserve to be investigated since according to Jawwa et al. and Kogon et al. (Jawa et al., 2021; Kogon et al., 2013) these are physical symptoms of depression. Depression can exacerbate renal failure symptoms, affects adherence to treatment, increases hospitalizations, morbidity, mortality (Liu et al., 2017; Treadwell, 2017), and suicide rate (Fonseca et al., 2022). This creates the need to investigate depression in adolescents with renal failure on dialysis, and for psychiatrists from the health services to systematically assess this problem in this group. Underestimated or untreated depression and/or anxiety can lead to adolescents’ decreased quality of life and may weaken their adherence to treatment and self-care (Hames et al., 2021; Miller et al., 2021).

Even though the present study did not investigate the quality of life, it was very evident in the adolescents’ reports life quality was severely deteriorated due to dialysis, an aspect that is similar to some studies (Belayev et al., 2015; Tjaden et al., 2016), which show the time spent on dialysis deteriorates the adolescents’ quality of life, as well as restrictions on water and food consumption and care with the dialysis catheter. Most of the adolescents in the present study reported they live only for the disease and its treatment, they could do nothing more than live “attached” to a machine, live with thirst and without being able to eat what they wanted, taking many medications and dedicated to keep medical appointments.

### Struggling with the maddening thirst experience

Regarding the restriction of fluid intake, the adolescents showed autonomy since they adjusted to their needs the recommendation to drink 800 cc of liquids a day. They considered that 800 cc of total water per day was a very low quantity, therefore, complying with this restriction was not possible; they believed they could exceed that amount without affecting their health. Indeed, for most young people, ingesting more than 800 cc of fluids did not cause clinical complications, which suggests that the recommendations on fluid intake could be adjusted to the individual needs of each person instead of the 800 cc. amount to everybody.

Some studies have shown living with fluid intake restriction is a dramatic daily battle, generating stress and suffering for youngsters with this health condition (Glyde et al., 2019; Porcu et al., 2007). There are motivational interventions (Oller et al., 2018) to help adolescents not to exceed their fluid intake, but the relevance of customizing the fluid volume a young person with kidney failure could drink has not been investigated. It is urgent to do studies in this sense, especially that show the need for this self-care practice,

Another high-priority issue on the agendas of researchers interested in the experiences of adolescents on dialysis is the strategies they use to better coexist with the restriction of daily fluids, such as drinking more fluids on the day of dialysis. Such strategies allow adolescents to cope with thirst more kindly, even for a limited time. Adolescents on dialysis have to go a long way to find some balance in their daily fluid intake and the health team has the opportunity to support them by recognizing their individuality and autonomy, which will help them to live better with the recommended restrictions.

### Deciding what, when and how much food to eat

The study adolescents’ everyday experiences in following the prescribed food intake are very similar to other findings; they reported few food options, technical difficulties in following the recommendations, sadness, anguish, and frustration when not being able to eat the desired foods. In general, individuals with ESRD on dialysis perceive their food options are very limited, to follow the prescribed diet is overwhelming, frustrating, emotionally demanding, complex, challenging, and produces anguish, sadness, and frustration (Lambert et al., 2017; Morris & Lycett, 2020).

In the study, adolescents learned to choose the foods to consume by trial and error, that is, they consumed non-recommended foods and verified whether or not their health was affected by them. This practice arose because the health team was not consistent in the recommendations and prohibitions; sometimes they prohibited certain foods and other times they recommended their consumption. The adolescents’ practices of “*Eating occasionally forbidden food” and “eating everything but few quantities”* have not been reported in other studies, but those should be studied to know their impact on the adolescents’ health and well-being.

It should be noted the lack of financial resources was an important determinant in adherence to the renal diet. Some adolescents had reduced possibilities of buying some of the recommended foods, which is also described in the literature (Roach et al., 2017). However, further studies are required on diet compliance in adolescents with kidney failure on dialysis from different social, economic, and ethnic groups to identify strategies that support their commitment to diet adherence. On the other hand, entities of the food industry must rethink their products and try to offer options for young people who suffer from chronic diseases such as kidney disease. All the strategies aimed at improving the diet of young people with so many restrictions contribute to their quality of life, increased adherence to nutritional recommendations, and could reduce morbidity and mortality rates in this population (Kelly et al., 2017).

### Taking care of an alien which helps to survive

The study adolescents were aware their survival required living with the dialysis accesses such as haemodialysis and peritoneal catheter and the arteriovenous fistula. Although the adolescents accepted these devices, those caused suffering from not being able to go to swimming pools and rivers, technical difficulties to bathe daily, discomfort to sleep, and social distancing due to fear of infections and the risk of displacement of the catheter. The adolescents also live with the shame caused by these grotesque body-image-impairing devices, which are an obstacle to relating to peers and wearing clothes according to preferences. This is similar to the findings in other study, especially for girls (Kelly et al., 2017).

There is a paucity of studies on the experiences of young people with dialysis devices. The literature usually reports about surgical techniques to place the catheters, the risk of infection, dressings to cover them, agents for cleaning and disinfection of the catheter entry holes among others issues. The scarcity of studies regarding the perspective of adolescents when living with a dialysis catheter denotes the great inclination towards biomedical aspects both in research and in the health care services, which leads to adolescent’s dissatisfaction due to lack of appropriateness of institutional discourses and services to the adolescent’s needs and expectations (Morales & Castillo, 2007).

Studies such as Wasse, Zhang, Johansen, and Kutner (Wasse et al., 2013) on the factors that contribute to low levels of physical activity among adolescents with kidney failure on dialysis concluded that dialysis catheters are the factor that is most strongly related to low level of physical activity. This finding coincides with the present study: adolescents never performed physical activity regularly and when they did, they always felt fear of infections and catheter displacement. It is important to take into account the adolescent’s need to have creative strategies that allow them to do physical activity since the benefits of physical exercise for adolescents with this health condition are well documented (Cardoso et al., 2019; Dastidehkordi et al., 2019).

The arteriovenous fistula was better accepted by the adolescents in the study, but it was still the stranger they had to take care of in order to survive. They felt discomfort and shame for having an “imperfect body” as other studies have reported (Richard & Engebretson, 2010; Silva et al., 2018). Self-care practices with the fistula varied particularly by the degree of the aneurysm; all took proper care of the skin covering the fistula, but not all followed the other recommendations for its care despite having received training and acknowledging that they had the necessary knowledge to adequately perform self-care. These findings are contrary to those of Pessoa and Linhares (Pessoa & Linhares, 2015), who reported a good attitude of young people towards self-care of arteriovenous fistula, lack of knowledge on the subject, and inadequate self-care of vascular access. The authors conclude that inadequate knowledge probably influenced inadequate self-care and recommend providing young people with written material since it allows users to read and re-read the information.

More studies are required to know how is for the adolescents to live every day with dialysis devices so their life quality and self-care could be improved thru health services based on their needs, concerns and practices.

### Taking the medicines when feeling in need of them

Lack of adherence to drug treatment in persons on permanent dialysis is a widely recognized and studied problem because it contributes significantly to excess morbidity and mortality in this population. The quantification of adherence shows dramatic and diverse figures from 3% to 80% (Schmid et al., 2009). The findings of the present study coincide with what has been published; most of the study adolescents did not take their medications according to the medical prescription; they decided which ones to take or not and when to take them. The decision was based on the fact they believed the medicines have many adverse effects and, in many cases cause more harm. The hierarchy established by the adolescents to take the medications was based on their previous experience regarding compliance taking the same medicine. They took anticonvulsants daily to avoid seizures that led them to the ICU, antihypertensives were taken if they perceived symptoms of increased blood pressure. They did not take vitamins and Calcitriol because they perceived those as unnecessary. Wileman et al. (Wileman et al., 2011) and Karamanidou, Clatworthy, Weinman and Horne (Karamanidou et al., 2008) consider adolescent’s beliefs are fundamental when taking medicines, and particularly when deciding which ones to take. These authors reported phosphorus binders as the main drugs not taken by patients, which differs from the findings of the present study.

Persons with ESRD on dialysis have the highest medicine burden reported in the literature for individuals with chronic disease (Chiu et al., 2009). Having to take a large amount of medication (up to 23 tablets) each day represents an overwhelming challenge for adolescents as the schedules were every two, three, or four hours. Research on the experiences of youngsters with pharmacological therapy should be increased; listening to their voices can help to identify unknown situations for the health professionals and offer care programs and services with creative and flexible strategies according to the adolescent’s particular needs. On the other hand, since self-care is dynamic over time, which makes it a fragile practice that requires great efforts from all to maintain and strengthen it and to achieve adequate health results in the short, medium, and long terms.

Illness and treatment changed how the study adolescents exist every day since their way of being was according to the illness and dialysis self-care demands. These demands made them to exist in the mode of being that Heidegger called present-at- hand, it is in a reflexive mode. They had to be aware of every daily moment in their live, they could not exist on the ready-to- hand mode as most people do it. They embody death as their possibility but they continue having dreams and hopes that motivate them to care for themselves every day.

The adolescents’ self-care practices mainly were done to preserve their health and manage dialysis and pharmacologic treatment demands. In other words, the adolescents did self-care activities not to get sick neither be hospitalized and get the kidney transplant.

## Conclusions

The adolescents perceived dialysis as *their option to stay alive*, it greatly disturbs the adolescent´s everyday world at home and outside since they should plan their life only in light of the dialysis regardless its modality; this situation produced a sense of slavery from dialysis in the adolescents. However, self-care practices show adherence among the study adolescents.

Self-care practices regarding fluid and eating restrictions were mostly based on the adolescent´s beliefs derived from their experiences when broken the guidelines and also by trial and error.

The self-care practices with the dialysis accesses were performed according to the health team guidelines mainly to avoid catheter infections and displacement.

The high number of prescribed medications also causes great disturbance in the adolescent´s daily life and increases the adolescents’ idea of slavery. Only the anticonvulsants were taken following the prescription but after having one seizure episode. Other medicines were taking as needed by the youngsters.

Adapting the self-care practices to their beliefs and habits allows the adolescents to live with dialysis in a less traumatic way. To guarantee the quality of care for adolescents with kidney failure on dialysis, the health care team must recognize and understand the adolescent´s individual needs so they feel recognized and willing to make agreements concerning the self-care needed for them. More studies are needed to better understand the adolescent´s world when being on dialysis.

## Recommendations for practice

The adolescents lived experiences with their self-care in this study suggest the health institutions and the kidney team should implement fundamental changes in the current clinical practice in caring for the adolescents in dialysis. The following changes are suggested as ways to move along the goal of health care based on the adolescent’s individuality and autonomy:

- Implement strategies that help the adolescents to perceive the uncertainty regarding the disease progression as an opportunity to improve their self-care, particularly to comply with the pharmacological treatment. Virtual media and written materials as well as peer groups could be useful since the adolescent love to be connected to internet and peers. Providing the scientific evidence that support the treatment also could be useful.
- Develop approaches to set joined goals regarding taking the medicines, food and fluids choices during the regular follow up appointment of the adolescents with the nephrologist, the nutritionist, and the nurses in charge of their care.
- Review the scientific evidence for food and fluid restrictions according to the dialysis modality to set specific guidelines for teens.
- Develop approaches that promote teens’ care of psychological conditions during and in between the follow appointments. Virtual media and peer groups could be used to carry out the activities.
- Stablish teen group care as a strategy to optimize the institutional resources. This approach has been used successfully in adults with chronic illness and pregnant women. This approach seems very appropriate for adolescents due to their need to interact with their peers. Groups could be organized according to the teen’s gender.

## Limitations

Although the findings of a phenomenological study are not intended to be generalized to the population, as is customary in quantitative studies with statistically representative samples, the results of the present study can be transferred to adolescents on dialysis in our country and other countries with similar contexts. A larger sample would have been ideal, but two issues impede to have a larger sample: 1) difficulty in obtaining the basic information to contact the adolescents, such as names, addresses and telephone numbers. The directors of the renal units, which are multinational companies, did not allow the researchers to have access to this data, 2) the fragile health conditions of some adolescents, that led to multiple and prolonged hospitalizations, many times forced to cancel and reschedule the interviews since the adolescents wanted to continue in the study.

## References

[cit0001] Adriaans, D. J., Dierick van Daele, A. T., van Bakel, M. J. H. M., Nieuwenhuijzen, G. A., Teijink, J. A., Heesakkers, F. F., & van Laarhoven, H. W. (2021). Digital self-management support tools in the care plan of patients with cancer: Review of randomized controlled trials. *Journal of Medical Internet Research*, 23(6), e20861. 10.2196/20861 34184997 PMC8278296

[cit0002] Belayev, L., Mor, M. K., Sevick, M. A., Shields, A. M., Rollman, B. L., Palevsky, P. M., Arnold, R. M., Fine, M. J., & Weisbord, S. D. (2015). Longitudinal associations of depressive symptoms and pain with quality of life in patients receiving chronic hemodialysis. *Hemodialysis International International Symposium on Home Hemodialysis*, 19(2), 216–13. 10.1111/hdi.12247 25403142 PMC4383700

[cit0003] Bikbov, B., Purcell, C. A., Levey, A. S., Smith, M., Abdoli, A., Abebe, M., Adebayo, O. M., Afarideh, M., Agarwal, S. K., Agudelo-Botero, M., Ahmadian, E., Al-Aly, Z., Alipour, V., Almasi-Hashiani, A., Al-Raddadi, R. M., Alvis-Guzman, N., Amini, S., Andrei, T., Andrei, C. L. … GBD Chronic Kidney Disease Collaboration. (2020). Global, regional, and national burden of chronic kidney disease, 1990–2017: A systematic analysis for the global burden of disease study 2017. *The Lancet*, 395(10225), 709–733. 10.1016/S0140-6736(20)30045-3 PMC7049905 32061315

[cit0004] Booster, G. D., Oland, A. A., & Bender, B. G. (2019). Treatment adherence in young children with asthma. *Immunology and Allergy Clinics of North America*, 39(2), 233–242. 10.1016/j.iac.2018.12.006 30954173

[cit0005] Bukhsh, A., Nawaz, M. S., Ahmed, H. S., & Khan, T. M. (2018). A randomized controlled study to evaluate the effect of pharmacist-led educational intervention on glycemic control, self-care activities and disease knowledge among type 2 diabetes patients: A consort compliant study protocol. *Medicine (Baltimore)*, 97(12), e9847. 10.1097/MD.0000000000009847 29561461 PMC5895327

[cit0006] Cardoso, R., Machado, A., Bueno, R., Bohlke, M., Oses, J. P., Boscolo, F., Correa, F., Gonzalez, M. C., Rombaldi, A. J., et al. (2019). Effects of continuous moderate exercise with partial blood flow restriction during hemodialysis: A protocol for a randomized clinical trial. *MethodsX*, 6, 190–198. 10.1016/j.mex.2019.01.005 30740314 PMC6357543

[cit0007] Chávez, J. S., García, G., & Lombardi, R. (2018). EpidemiologíEpidemiologíA y desenlaces de la lesión renal aguda en latinoamérica. *Gaceta Medica de Mexico*, 154(1), S6–14. 10.24875/GMM.M18000067 30074021

[cit0008] Chiu, Y. W., Teitelbaum, I., Misra, M., de Leon EM, Adzize, T., Mehrotra, R., & de Leon, E. M. (2009). Pill burden adherence, hyperphosphatemia, and quality of life in maintenance dialysis patients. *Clinical Journal of the American Society of Nephrology : CJASN*, 4(6), 1089–1096. 10.2215/CJN.00290109 19423571 PMC2689877

[cit0009] Chou, H. H., Chiou, Y. -Y., Chiou, Y. -H., Tain, Y. -L., Wang, H. -H., Yu, M. -C., Hsu, C. -C., & Lin, C. -Y. (2018). Mortality risks among various primary renal diseases in children and adolescents on chronic dialysis. *Journal of Clinical Medicine*, 7(11), 414. 10.3390/jcm7110414 30400589 PMC6262556

[cit0010] Chua, A. N., & Warady, B. A. (2011). Adherence of pediatric patients to automated peritoneal dialysis. *Pediatric Nephrology (Berlin, Germany)*, 26(5), 789–793. 10.1007/s00467-011-1792-2 21350797

[cit0011] Dashtidehkordi, A., Shahgholian, N., & Attari, F. (2019). Exercise during hemodialysis and health promoting behaviors: A clinical trial. *BMC Nephrology*, 20(1), 96. 10.1186/s12882-019-1276-3 30890122 PMC6425622

[cit0012] Fernandes, L., Nóbrega, V., Silva, M., Machado, A., & Collet, N. (2017). Supported self-care for children and adolescents with chronic disease and their families. *Revista Brasileira de Enfermagem*, 70(6), 1318–1329. 10.1590/0034-7167-2016-0553 29160496

[cit0013] Fleeman, N., & Bradley, P. M. (2018). Care delivery and self-management strategies for children with epilepsy. *Cochrane Database Syst Rev*, 3(3). CD006245. 10.1002/14651858.CD006245.pub4. PMC6494414 29493780

[cit0014] Fonseca, E., Al-Halabí, S., Pérez, A., & Debbané, M. (2022). Risk and protective factors in adolescent suicidal behaviour: A network analysis. *International Journal of Environmental Research and Public Health*, 19(3), 1784. 10.3390/ijerph19031784 35162805 PMC8834911

[cit0015] Gauci, J., Bloomfield, J., Lawn, S., Towns, S., & Steinbeck, K. (2021). Effectiveness of self-management programmes for adolescents with a chronic illness: A systematic review. *Journal of Advanced Nursing*, 77(9), 3585–3599. 10.1111/jan.14801 33630315

[cit0016] Glyde, M., Keane, D., Dye, L., & Sutherland, E. (2019). Patients’ perceptions of their experience, control and knowledge of fluid management when receiving haemodialysis. *Journal of Renal Care*, 45(2). 10.1111/jorc.12275 30938066

[cit0017] Grewal, M. K., Mehta, A., Chakraborty, R., & Raina, R. (2020). Nocturnal home hemodialysis in children: Advantages, implementation, and barriers. *Seminars in Dialysis*, 33(2), 109–119. 10.1111/sdi.12863 32155297

[cit0018] Guest, G., Namey, E., & Chen, M. (2020). A simple method to assess and report thematic saturation in qualitative research. *Plos One*, 15(5), e0232076. 10.1371/journal.pone.0232076 32369511 PMC7200005

[cit0019] Hames, A., Matcham, F., Makin, I., Day, J., Joshi, D., & Samyn, M. (2021). Adherence mental health and illness perceptions in autoimmune liver disease: Looking beyond liver function tests. *Journal of Pediatric Gastroenterology and Nutrition*, 73(3), 376–384. 10.1097/MPG.0000000000003119 33720085

[cit0020] Jawa, N. A., Rapoport, A., Widger , K., Zappitelli, M., Davison, S., Jha, S., Dart, A., Matsuda, M., et al. (2021). Development of a patient-reported outcome measure for the assessment of symptom burden in pediatric chronic kidney disease (PRO-Kid). *Pediatric Nephrology (Berlin, Germany)*, 10, 1–10. 10.1007/s00467-021-05269-4 PMC8579900 34761300

[cit0021] Jose, J., & Bond, C. (2021 17). Medication adherence: Still a problem. *The International Journal of Pharmacy Practice*, 29(2), 93–95. 10.1093/ijpp/riaa019 33729522

[cit0022] Karamanidou, C., Clatworthy, J., Weinman, J., & Horne, R. (2008). A systematic review of the prevalence and determinants of nonadherence to phosphate binding medication in patients with end-stage renal disease. *BMC Nephrology*, 9(1), 2. 10.1186/1471-2369-9-2 18237373 PMC2270809

[cit0023] Kelly, J. E. A., Palmer, S. C., Wai, S. N., Ruospo, M., Carrero, J. -J., Campbell, K. L., & Strippoli, G. F. M. (2017). Healthy dietary patterns and risk of mortality and ESRD in CKD: A meta-analysis of cohort studies. *Clinical Journal of the American Society of Nephrology : CJASN*, 12(2), 272–279. 10.2215/CJN.06190616 27932391 PMC5293335

[cit0024] Kim, S. (2018). Development and effect of a rational-emotive-behaviour-therapy-based self-management programme for early renal dialysis patients. *Journal of Clinical Nursing*, 27(21–22), 4179–4191. 10.1111/jocn.14608 29968272

[cit0025] Kogon, A. J., Kim, J. Y., Laney, N., Radcliffe, J., Hooper, S. R., Furth, S. L., & Hartung, E. A. (2019). Depression and neurocognitive dysfunction in pediatric and young adult chronic kidney disease. *Pediatric Nephrology (Berlin, Germany)*, 34(9), 1575–1582. 10.1007/s00467-019-04265-z 31049719

[cit0026] Kogon, A. J., Vander Stoep, A., Weiss, N. S., Smith, J., Flynn, J. T., & McCauley, E. (2013). Depression and its associated factors in pediatric chronic kidney disease. *Pediatric Nephrology*, 28(9), 1855–1861. 10.1007/s00467-013-2497-5 23700174 PMC3897296

[cit0027] Krockow, E. M., Riviere, E., & Frosch, C. A. (2019). Improving shared health decision making for children and adolescents with chronic illness: A narrative literature review. *Patient Education and Counseling*, 102(4), 623–630. 10.1016/j.pec.2018.11.017 30578102

[cit0028] Lambert, K., Mullan, J., & Mansfield, K. (2017). An integrative review of the methodology and findings regarding dietary adherence in end stage kidney disease. *BMC Nephrology*, 18(1), 318. 10.1186/s12882-017-0734-z 29061163 PMC5653982

[cit0029] Leonard, V. (1994). *A heideggerian phenomenological perspective on the concept of person. en: Benner, editora. P interpretive phenomenology: embodiment, caring, and ethics in health and illnes*. SAGE Publications Inc.

[cit0030] Liu, C. H., Yeh, M. K., Weng, S. C., Bai, M. Y., & Chang, J. C. (2017 1). Suicide and chronic kidney disease: A case-control study. *Nephrology, Dialysis, Transplantation : Official Publication of the European Dialysis and Transplant Association - European Renal Association*, 32(9), 1524–1529. 10.1093/ndt/gfw244 27638910

[cit0031] Marani, H., Fujioka, J., Tabatabavakili, S., & Bollegala, N. (2020). Systematic narrative review of pediatric-to-adult care transition models for youth with pediatric-onset chronic conditions. *Children and Youth Services Review*, 118(2), 105415. 10.1016/j.childyouth.2020.105415

[cit0032] Mayan, M., & Introductoria, N. (2001). *Una introducción a los métodos cualitativos. Módulo de entrenamiento para estudiantes y profesionales*. International Institute for Qualitative Methodology.

[cit0033] Miller, L., Campo, J. V., & Ropper, A. H. (2021). Depression in adolescents. *The New England Journal of Medicine*, 385(5), 445–449. 10.1056/NEJMra2033475 34320289

[cit0034] Miyata, K., Shen, J. I., Nishio, Y., Haneda, M., Dadzie, K. A., Sheth, N. R., Kuriyama, R., Matsuzawa, C., Tachibana, K., Harbord, N. B., & Winchester, J. F. (2018). Patient knowledge and adherence to maintenance hemodialysis: An international comparison study. *Clinical and Experimental Nephrology*, 22(4), 947–956. 10.1007/s10157-017-1512-8 29185127 PMC5972057

[cit0035] Modi, Z. J., Lu, Y., Ji, N., Kapke, A., Selewski, D. T., Dietrich, X., Abbott, K., Nallamothu, B. K., Schaubel, D. E., Saran, R., & Gipson, D. S. (2019). Risk of cardiovascular disease and mortality in young adults with end-stage renal disease: An analysis of the US renal data system. *JAMA Cardiol*, 4(4), 353–362. 10.1001/jamacardio.2019.0375 30892557 PMC6484951

[cit0036] Morales, L. C., & Castillo, E. (2007). Vivencias de los (as) adolescentes en diálisis: Una vida con múltiples pérdidas, pero con esperanza. *Colombia Médica*, 38(4), 44–53.

[cit0037] Morris, A., & Lycett, D. (2020). Experiences of the dietary management of serum potassium in chronic kidney disease: Interviews with UK adults on maintenance hemodialysis. *Journal of Renal Nutrition : The Official Journal of the Council on Renal Nutrition of the National Kidney Foundation*, 30(6), 556–560. 10.1053/j.jrn.2020.01.025 32273228

[cit0038] Nsiah, I., Imeri, H., Jones, A. C., Bentley, J. P., Barnard, M., & Kang, M. (2021). The impact of medication synchronization programs on medication adherence: A meta-analysis. *Journal of the American Pharmaceutical Association*, 61(4), e202–211. 10.1016/j.japh.2021.02.005 33741277

[cit0039] Oller, G., Oliveira, M. P., Cesarino, C. B., Teixeira, C. R., Costa, J. A., & Kusumota, L. (2018). Clinical trial for the control of water intake of patients undergoing hemodialysis treatment. *Revista Latino-Americana de Enfermagem*, 26, e3091. 10.1590/1518-8345.2694.3091 30517579 PMC6280168

[cit0040] Ozen, N., Cinar, F. I., Askin, D., Mut, D., & Turker, T. (2019). Nonadherence in hemodialysis patients and related factors: A multicenter study. *The Journal of Nursing Research : JNR*, 27(4), e36. 10.1097/jnr.0000000000000309 30720548 PMC6641098

[cit0041] Pessoa, N., & Linhares, F. (2015). Pacientes em hemodiálise com fístula arteriovenosa: Conhecimento, atitude e prática. Escola Anna Nery, 19(1), 73–79. 10.5935/1414-8145.20150010

[cit0042] Porcu, M., Fanton, E., & Zampieron, A. (2007). Thirst distress and interdialytic weight gain: A study on a sample of haemodialysis patients. *Journal of Renal Care*, 33(4), 179–181. 10.1111/j.1755-6686.2007.tb00069.x 18298036

[cit0043] Richard, C. J., & Engebretson, J. (2010). Negotiating living with an arteriovenous fistula for hemodialysis. *Nephrology Nursing Journal : Journal of the American Nephrology Nurses’ Association*, 37(4), 363–374 quiz 375. PMID: 20830944. 20830944

[cit0044] Roach, L. A., Lambert, K., Holt, J. L., & Meyer, B. J. (2017). Diet quality in patients with end-stage kidney disease undergoing dialysis. *Journal of Renal Care*, 43(4), 226–234. 10.1111/jorc.12215 28944596

[cit0045] Schmid, H., Hartmann, B., & Schiffl, H. (2009). Adherence to prescribed oral medication in adult patients undergoing chronic hemodialysis: A critical review of the literature. *European Journal of Medical Research*, 14(5), 185–190. 10.1186/2047-783x-14-5-185 19541573 PMC3351975

[cit0046] Shorey, S., & ED, N. (2020). The lived experiences of children and adolescents with non-communicable disease: A systematic review of qualitative studies. *Journal of Pediatric Nursing*, 51, 75–84. 10.1016/j.pedn.2019.12.013 31926405

[cit0047] Silva, D., Silva, R., Pereira, E., Ferreira, H., Alcantara, V., & Oliveira, F. (2018). The body marked by the arteriovenous fistula: A phenomenological point of view. *Revista Brasileira de Enfermagem*, 71(6), 2869–2875. 10.1590/0034-7167-2017-0898 30517387

[cit0048] Slater, H., Campbell, J. M., Stinson, J. N., Burley, M. M., & Briggs, A. M. (2017). End user and implementer experiences of mHealth technologies for noncommunicable chronic disease management in young adults: Systematic review. *Journal of Medical Internet Research*, 19(12), e406. 10.2196/jmir.8888 29233804 PMC5743925

[cit0049] Smith, J. A., & Shinebourne, P. Interpretative phenomenological analysis. In C. H. C. PM, D. Long, A. Panter, D. Rindskopf, & K. Sher Eds. *APA handbook of research methods in psychology, Vol. 2. Research designs: Quantitative, qualitative, neuropsychological, and biological*pp. 73–82. American Psychological Association. 10.1037/13620-005

[cit0050] Tan, Q. E. C., Gao, X., Ang, W. H. D., & Lau, Y. (2021). Medication adherence: A qualitative exploration of the experiences of adolescents with systemic lupus erythematosus. *Clinical Rheumatology*, 40(7), 2717–2725. 10.1007/s10067-021-05583-0 33566194

[cit0051] Tjaden, L., Grootenhuis, M., Noordzij, M., & Groothoff, J. (2016). Health-related quality of life in patients with pediatric onset of end-stage renal disease: State of the art and recommendations for clinical practice. *Pediatric Nephrology (Berlin, Germany)*, 31(10), 1579–1591. 10.1007/s00467-015-3186-3 26310616 PMC4995226

[cit0052] Treadwell, A. A. (2017). Examining depression in patients on dialysis. *Nephrology Nursing Journal : Journal of the American Nephrology Nurses’ Association*, 44(4), 295–307 PMID: 29160964. 29160964

[cit0053] Tuohy, D., Cooney, A., Dowling, M., Murphy, K., & Sixsmith, J. (2013). An overview of interpretive phenomenology as a research methodology. *Nurse Researcher*, 20(6), 17–20. 10.7748/nr2013.07.20.6.17.e315 23909107

[cit0054] Vasileiou, K., Barnett, J., Thorpe, S., & Young, T. (2018). Characterising and justifying sample size sufficiency in interview-based studies: Systematic analysis of qualitative health research over a 15-year period. *BMC Medical Research Methodology*, 18(1), 148. 10.1186/s12874-018-0594-7 30463515 PMC6249736

[cit0055] Wasse, H., Zhang, R., Johansen, K. L., & Kutner, N. (2013). ESRD patients using permanent vascular access report greater physical activity compared with catheter users. *International Urology and Nephrology*, 45(1), 199–205. 10.1007/s11255-012-0137-9 22350837 PMC3467352

[cit0056] Wileman, V., Chilcot, J., Norton, S., Hughes, L., Wellsted, D., & Farrington, K. (2011). Choosing not to take phosphate binders: The role of dialysis patients’ medication beliefs. *Nephron Clinical Practice*, 119(3), c205–213. 10.1159/000329106 21832846

[cit0057] World Health Organization [Internet]. 2014 [Consultado el 27 de febrero 2022]‎. Toolkit for delivering the 5a’s and 5r’s brief tobacco interventions in primary care. Disponible en: https://apps.who.int/iris/handle/10665/112835.

